# Placing a Price on Medical Device Innovation: The Example of Total Knee Arthroplasty

**DOI:** 10.1371/journal.pone.0062709

**Published:** 2013-05-06

**Authors:** Lisa G. Suter, A. David Paltiel, Benjamin N. Rome, Daniel H. Solomon, Thomas S. Thornhill, Stanley K. Abrams, Jeffrey N. Katz, Elena Losina

**Affiliations:** 1 Department of Medicine, Yale School of Medicine, New Haven, Connecticut, United States of America; 2 Department of Medicine, VA Connecticut Healthcare System, West Haven, Connecticut, United States of America; 3 Department of Health Policy and Management, Yale School of Public Health, New Haven, Connecticut, United States of America; 4 Department of Orthopedic Surgery, Orthopedics and Arthritis Center for Outcomes Research, Brigham & Women’s Hospital, Boston, Massachusetts, United States of America; 5 Section of Clinical Sciences, Division of Rheumatology, Immunology and Allergy, Department of Medicine, Brigham & Women’s Hospital, Boston, Massachusetts, United States of America; 6 Harvard Medical School, Boston, Massachusetts, United States of America; 7 Department of Orthopedic Surgery, Brigham & Women’s Hospital, Boston, Massachusetts, United States of America; 8 Department of Epidemiology, Harvard School of Public Health, Boston, Massachusetts, United States of America; 9 Department of Biostatistics, Boston University School of Public Health, Boston, Massachusetts, United States of America; Groningen Research Institute of Pharmacy, United States of America

## Abstract

**Background:**

Total knee arthroplasty (TKA) is common, effective, and cost-effective. Innovative implants promising reduced long-term failure at increased cost are under continual development. We sought to define the implant cost and performance thresholds under which innovative TKA implants are cost-effective.

**Methods:**

We performed a cost-effectiveness analysis using a validated, published computer simulation model of knee osteoarthritis. Model inputs were derived using published literature, Medicare claims, and National Health and Nutrition Examination Survey data. We compared projected TKA implant survival, quality-adjusted life expectancy (QALE), lifetime costs, and cost-effectiveness (incremental cost-effectiveness ratios or ICERs) of standard versus innovative TKA implants. We assumed innovative implants offered 5–70% decreased long-term TKA failure rates at costs 20–400% increased above standard implants. We examined the impact of patient age, comorbidity, and potential increases in short-term failure on innovative implant cost-effectiveness.

**Results:**

Implants offering ≥50% decrease in long-term TKA failure at ≤50% increased cost offered ICERs <$100,000 regardless of age or baseline comorbidity. An implant offering a 20% decrease in long-term failure at 50% increased cost provided ICERs <$150,000 per QALY gained only among healthy 50–59-year-olds. Increasing short-term failure, consistent with recent device failures, reduced cost-effectiveness across all groups. Increasing the baseline likelihood of long-term TKA failure among younger, healthier and more active individuals further enhanced innovative implant cost-effectiveness among younger patients.

**Conclusions:**

Innovative implants must decrease actual TKA failure, not just radiographic wear, by 50–55% or more over standard implants to be broadly cost-effective. Comorbidity and remaining life span significantly affect innovative implant cost-effectiveness and should be considered in the development, approval and implementation of novel technologies, particularly in orthopedics. Model-based evaluations such as this offer valuable, unique insights for evaluating technological innovation in medical devices.

## Introduction

Many medical devices are marketed with claims of reduced long-term device failure and/or adverse events. Such claims are often based upon laboratory or *ex vivo* data extrapolated to long-term outcomes in humans and cannot be evaluated with short-term trials. Given the costs and limitations of prospectively collecting long-term efficacy data on innovative devices, simulation modeling is an ideal methodology to evaluate current technologies and anticipate potential health and economic consequences of future technologies.

Total knee arthroplasty (TKA) implant innovation provides an informative case study for examining the impact of medical device innovation on healthcare costs [1]. Over 620,000 TKAs were performed in the US in 2009, predominantly for end-stage osteoarthritis (OA) [2]. TKA inpatient costs exceed $9 billion per year, the highest aggregate cost among the 10 fastest growing procedures [3]. Over 85% of recipients have functional improvement after TKA [4]–[7], and TKA is highly cost-effective [8].

Fueled by this success and trends suggesting that TKA recipients are younger, heavier, more active, and surviving longer after TKA than previously [2], [9]–[11], over 35 TKA systems or components have been approved by the Food and Drug Administration (FDA) each year for the last decade through the 510(k) clearance process [12]. This process allows approval of perceived low-risk medical devices based upon claims of similarity to existing approved devices and likely incentivizes incremental improvements. Indeed, most claims of improved device durability are based upon *ex vivo* studies [13]–[19], rather than clinical trials, and such innovations come at a price, as newer implants are more costly [20]–[22].

Trade-offs between incremental improvements and their costs are poorly understood. With rising healthcare costs, it is critical to examine the value of technological advancements over existing devices. TKA is an ideal example for studying the cost/benefit trade-offs for medical devices, particularly those that offer quality of life improvement rather than immediate survival benefit. Given the costs and limitations of prospectively collecting long-term efficacy data on innovative medical devices, such as TKA implants, simulation modeling is an ideal methodology to evaluate current technologies and anticipate potential health and economic consequences of future technologies.

We sought to examine cost/benefit trade-offs and present the long-term economic implications associated with the use of innovative TKA implants using a simulation modeling approach. Specifically, we sought to define the conditions, including the patient characteristics, implant performance, and cost assumptions, under which innovative TKA implants might offer clinical benefit and good value for additional dollars spent versus those scenarios under which innovative implants would result in greater costs and/or unfavorable economic value. More broadly, our analysis provides a paradigmatic case study of the impact of medical device innovation on clinical efficacy and cost-effectiveness.

## Methods

### Ethics Statement

The study was approved by the Institutional Review Board of Brigham & Women’s Hospital. Our research was conducted entirely in the U.S.A. No participants were involved and so no consent was required.

### Analytic Overview

We used a validated, previously published computer simulation model of knee OA natural history and management (the Osteoarthritis Policy or OAPol model) [23], [24] to forecast surgical, clinical and economic outcomes associated with “innovative” TKA implants (e.g., highly crossed-linked polyethylene or other innovative biomaterials) as compared with “standard” implants (i.e., an ultra-high molecular weight all polyethylene tibial component) [25] in persons with end-stage knee OA. Laboratory data indicate these newer implants experience less wear, implying greater durability [14], [19]. Our primary analysis varied implant durability and cost among different patient cohorts. Incremental cost-effectiveness ratios (ICERs), defined as the incremental benefits in additional quality-adjusted life expectancy (QALE) divided by the incremental costs, were estimated for innovative compared with standard implants.

### OAPol Model

The OAPol model is a state-transition simulation model that uses a series of annual transitions between health states to describe the natural history and clinical management of knee OA. Pain, comorbidity, treatment, quality of life, and resource use are used to define discreet health states. These characteristics also influence further disease progression, response to treatments, development of additional comorbidities, and mortality. The model is implemented as a Monte Carlo simulation, meaning a random number generator and a set of estimated probabilities are used to determine the state-to-state pathway followed by each simulated patient. Each patient’s course is then tracked until death, and large numbers of individual simulations are combined to estimate population outcomes and costs. This model has been used to estimate TKA cost-effectiveness [8], knee OA burden [24], and quality-adjusted life-years (QALYs) lost due to obesity and knee OA [23]. Model specifications, validation, and data sources have been previously published [1], [23] and are supplemented in the Technical Appendix S1.

### Study Population

We considered a population of individuals with end-stage (Kellgren-Lawrence grade IV) symptomatic knee OA at baseline. We considered four separate age and comorbidity cohorts to explore the impact of these characteristics on implant cost effectiveness. The four cohorts included 50–59 year-olds with and without comorbidity at the time of TKA and 70–79 year-olds with and without comorbidity at the time of TKA (see Population Demographics below for further explanation). We selected these age stratifications because while, historically, most US TKA recipients were in their 70 s, individuals under 65 years old represent the most rapidly growing group among TKA recipients [11].

### Input Parameters

Model input parameters are listed in Table 1 and summarized below. Further details are also provided in the Technical Appendix S1.

**Table 1:** Model Input Parameters*.

Parameter	Estimates							Source
**Quality of Life (in QALYs)**								
	**<65 years old**				**65+ years old**			
	**Healthy**	**With Comorbidity**			**Healthy**		**With Comorbidity**	
**Knee Pain -** *(No pain relief from TKA)*	0.806	0.679			0.884		0.757	NHANES 2005–2008 [33], [34], Losina et al, 2011
**No Knee Pain -** *(Pain relief from TKA)* †	0.952	0.867			0.943		0.858	[23]
**Surgical AE** ‡	0.60							Fisman et al, 2001 [32]
**Medical AE**	**Pneumonia**		**Myocardial Infarction**			**Pulmonary Embolism**		
	0.60		0.65			0.50		Taylor et al, 2009 [29],Melinkow et al, 2008 [30], Weaver et al, 2001 [31]
**Costs (in 2010 US$)**								
	**Healthy**				**With Comorbidity**			
**Underlying Medical Costs**	986–1,205				2,388–2,860			Lee et al, 2001 [35], NHANES 2005–2008 [33], [34]
**Analgesic Treatment** §	333							Red Book 2008 [36]
**TKA Costs**	**Primary TKA**				**Revision TKA**			
** First Year**	23,903				28,195			Losina et al, 2009 [8]
** Implant**	5,414							Mendenhall, 2004 [37]
** Annual Follow-Up**	103							CMS|| 2008 [39], Teeny et al, 2003 [40]
** Surgical AE**	21,213							HCUP 2008 [2]
** Medical AE**	**Pneumonia**			**Myocardial Infarction**		**Pulmonary Embolism**		
	10,388			18,478		12,090		HCUP 2008 [2]
	**∼ ** ***Standard Implants*** ** ∼**							
**TKA Efficacy & Adverse Events**								
	**Short-Term** ¶				**Long-Term** **			
	**Primary TKA**	**Revision TKA**			**Primary TKA**		**Revision TKA**	
**Technical Failure** †† **%**	1.1	1.1			1.36		1.36	Losina et al, 2009 [8]
**Pain Relief Success** ‡‡ **%**	86.2	74.3			96.0		94.4	Katz et al, 2007 [7]
**Adverse Events** §§ **%**								
** Surgical**	0.37	0.67			−		−	
** Medical**								
** Pneumonia**	1.27	1.27			−		−	
** Myocardial Infarction**	0.75	0.75			−		−	Katz et al, 2004 [27]
** Pulmonary Embolism**	0.74	0.74			−		−	
**Death from AE**	0.63	0.96			−		−	
	***∼ Innovative Implants ∼***							
**TKA Efficacy**								
	**Short-Term**				**Long-Term**			
**Technical Failure % (Range)**	1.1 (1.1–5.5)				0.4–1.1***			Losina et al, 2009 [8]
**Pain Relief Success % (Range)**	86.2 (75.9–100)				96.0			Katz et al, 2007 [7]
**Costs (in 2010 US$)**								
**TKA Costs**								
** First Year**	24,986–45,559							Losina et al, 2009 [8]
** Implant** †††	6,497–27,070							Mendenhall, 2004 [37]

* Further input parameters are provided in the online Technical Appendix and published literature (Suter 2011, Losina 2011).

† In order to capture significant pain, we defined pain as those individuals noting functional limitations. Quality of life estimates were derived from general population data, not specifically from TKA recipients.

‡ Derived from data for quality of life in persons with prosthetic joint infection.

§ Annual cost of analgesic treatment was added for individuals who did not achieve pain relief from their TKA.

|| CMS = Centers for Medicare and Medicaid Services.

¶ Short-term refers to the first year following primary or revision TKA.

** Long-term refers to the annual proportion of individuals experiencing a TKA outcome or AE each year following the first year after primary or revision TKA.

†† Technical failure is the percentage of individuals who required or qualified for revision surgery due to mechanical failure of the prosthesis, with or without associated symptoms.

‡‡ Pain relief success is the percentage of individuals achieving pain relief without technical failure. In instances of technical failure, pain relief was significantly less than the values reported above.

§§ Adverse events (AEs) only occurred in the first year after primary or revision TKA.

*** These values represent the range of failure rates used in the Primary Analysis (20–70% reductions in failure for innovative compared to standard implants). Standard implants had long-term failure rates as high as 5.44% in the sensitivity analysis exploring the impact of higher long-term failure in healthy 50–59-year-olds, producing a failure rate of 4.35% for an innovative implant offering a 20% decrease in long-term failure.

††† The implant cost is included in the total first-year cost of TKA.

#### Population demographics

We derived baseline distributions of sex, race, and ethnicity from 2000 Medicare claims data [26] and validated these parameters against a nationally representative sample of TKR recipients [2]. We dichotomized the baseline presence of medical comorbidities as “healthy” (no comorbidities) versus “with comorbidity” (including baseline obesity, cardiovascular disease, and other, non-OA musculoskeletal disorders). Cohorts “with comorbidity” experienced higher mortality, reduced quality of life, and increased underlying medical costs, compared to “healthy” cohorts.

#### Surgical outcomes: TKA Efficacy and Adverse Events for Standard Implants

Surgical outcomes consisted of TKA efficacy and adverse event (AE) rates which were derived from published estimates [7], [8], [27]. Standard TKA efficacy was comprised of pain relief efficacy (defined as the percentage of individuals achieving pain relief) and the absence of technical failure (defined as the percentage of individuals requiring or qualifying for revision surgery due to mechanical failure). Experiencing recurrent knee pain following surgery resulted in a decrement in quality of life as noted below. Both pain relief and technical failure were further divided into first year (“short-term”) versus subsequent year (“long-term”) rates.

The primary clinical outcome in the model consisted of QALE, a patient-relevant outcome assessment validated for use in a wide range of clinical conditions, including knee osteoarthritis and individuals undergoing TKA. QALE represents an ideal metric for assessing long-term outcomes following TKA as this procedure is focused primarily on improving function and reducing pain, thus directly affecting quality of life**.** Scores from the SF-36 questionnaire were transformed into quality of life weights using published methods [8], [28]. Quality of life estimates for TKA-related AEs were derived from published literature [29]–[32]. Quality of life valuations over time were influenced by the presence of pain, body mass index (obese or non-obese) and number of comorbidities (0–1, 2–3, or >3).

#### Economic outcomes: Costs

Underlying annual medical costs, in 2010 US dollars, were stratified by age, presence of knee pain and comorbidity and ranged from $986 to $2,860 [33]–[35]. An additional annual cost of $333 was added for individuals for whom TKA did not provide pain relief to account for analgesic treatment [36].

The cost of TKA (including hospital, physician, and rehabilitation costs) was estimated at $23,903 [8], of which the implant cost represented $5,414, based on survey data from a national sample of hospitals [37]. This estimate of implant cost is consistent with the findings of a recent study utilizing data from sixty-one hospitals in 2008 [38]. Annual follow-up cost for TKA (including a physician visit and imaging) was determined from Medicare reimbursement data and a national survey of orthopedic surgeons [39], [40].

### Primary Analysis

We measured comparative value in constant (2010) US dollars per QALY gained and reported all economic outcomes from a modified societal perspective, adjusted for time value (with 3% annual discounting), but excluding indirect costs [41]. We estimated the proportion of each cohort surviving with their original implant intact at 5, 10, 15, and 20 years after TKA. We calculated the QALE and costs associated with each combination of innovative TKA implant cost (20–400% over standard cost) [21], [37], [42] and reduction in long-term implant failure rate (5–70% decreases compared to standard implants) [14], [19] in each of the four primary patient cohorts. ICERs compared to standard TKA were calculated for each combination of implant cost, long-term implant failure rate, and patient cohort.

### Sensitivity Analyses

Based upon recent data suggesting that younger TKA recipients experience higher revision rates than older individuals [43], presumably due to higher activity levels leading to higher wear, we examined the impact of higher annual TKA failure rates (2.72–5.44%, versus 1.36% baseline risk) among the healthy 50–59-year-old cohort. We also examined the impact of increasing short-term TKA failure consistent with recent orthopedic device failures (100–500% increases in first-year technical failure) [44] while simultaneously decreasing long-term failure (5–70%), as one might anticipate from an innovative technology offering greater long-term benefit at higher short-term risk. We examined the impact of varying the proportion of patients achieving pain relief from innovative TKA implants; we considered values ranging from an 8% decrease to a 20% increase, compared to standard TKA. We also varied TKA cost [8], TKA offer and acceptance rates, and recipient demographics.

## Results

### Effect of Innovative Implants on Patient and Implant Survival

**Figure 1:**
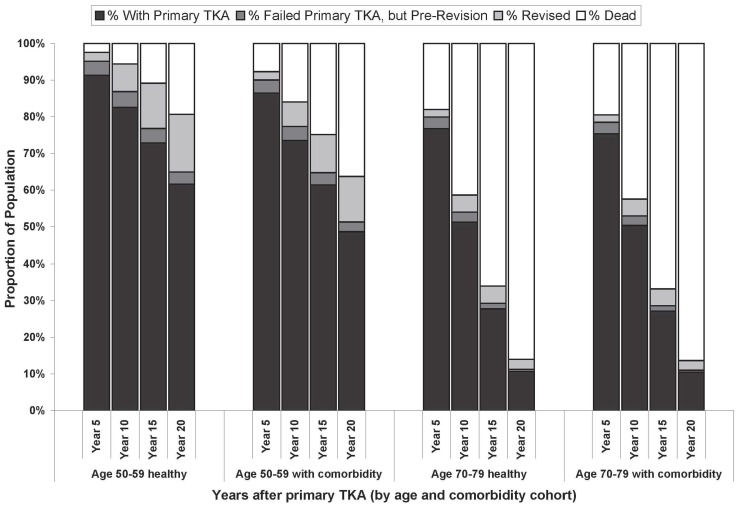
Proportion of population alive with original implant after Standard TKA by age and comorbidity. The estimated proportion of the population surviving with a successful primary TKA implant are noted by black bars among each of the four cohorts at 5, 10, 15, and 20 years after standard TKA. The dark grey bars represent the proportion of the population who have experienced technical TKA failure but have not undergone revision either due to the fact that their failure has not been observed by either the patient or their physician or they have been offered revision but refused. The light grey bars represent the proportion that has undergone revision TKA. As one moves from healthy 50–59-year-olds on the left to those with baseline comorbidity to healthy 70–79-year-olds and finally 70–79-year-olds with comorbidity on the far right, fewer individuals survive to experience TKA failure and therefore TKA revision. TKA = Total knee arthroplasty.

Patient survival 20 years following TKA ranged from 80.7% among healthy 50–59-year-olds to 13.6% among 70–79-year-olds with baseline comorbidity. Among healthy 50–59-year-olds, 64.9% survived and retained their primary TKA implant at 20 years (Figure 1), compared to 11.0% of 70–79-year-olds with baseline comorbidity. In total, 17.8% of healthy 50–59-year-olds and 9.4% of 70–79-year-olds with baseline comorbidity underwent revision surgery within 20 years of their primary TKA.

Innovative implants offering a 5% reduction in long-term TKA failure decreased the proportion of TKA recipients undergoing subsequent revision by only 0.79% and 0.39% (absolute percentage points) over 20 years in healthy 50–59-year-olds and 70–79-year-olds with comorbidity, respectively. Implants offering a 40% decrease in long-term TKA failure decreased the percent of TKA recipients undergoing revision by 6.29% among healthy 50–59-year-olds and 3.28% among 70–79-year-olds with comorbidity. An implant reducing long-term failure by 70% decreased revisions by 11.32% among healthy 50–59-year-olds and 5.83% among 70–79-year-olds with comorbidity.

### Effect of Innovative Implants on QALE, Costs, and Cost-Effectiveness

Remaining QALE and costs for standard and innovative implants are reported in Table 2. QALE after standard TKA ranged from 7.57 QALYs among 70–79-year-olds with baseline comorbidity to 16.43 QALYs among healthy 50–59-year-olds. Innovative TKA implants decreasing long-term TKA failure by 5% offered, on average, ≤0.01 additional QALYs (<four days) compared to standard TKA among all four populations. Implants decreasing long-term failure by 40% resulted in ≤0.06 additional QALYs among 50–59-year-olds and 0.02 additional QALYs among 70–79-year-olds. A 70% decrease in long-term failure offered an average of 0.10 additional QALYs (<37 quality-adjusted days) across the cohorts.

**Table 2:** Lifetime QALE, cost, and cost-effectiveness estimates associated with Standard and Innovative TKA.

				Ages 50–59							Ages 70–79								
% Decreasein Long-	% Increasein Implant			Healthy*			With comorbidity†				Healthy*				With comorbidity†				
Term Failure	Cost		Cost		QALE	ICER‡		Cost	QALE	ICER‡		Cost	QALE	ICER‡		Cost	QALE	ICER‡	
**Standard TKA**			$61,589		16.43	−		$82,523	12.88	−		$42,186	8.57	−		$55,606	7.57	−	
		**20%**	$62,508		16.44	$98,576		$83,443	12.88	$256,918		$43,152	8.57	$285,846		$56,568	7.58	$253,792	
		**50%**	$64,135		16.44	$272,947		$85,042	12.88	$703,083		$44,734	8.57	$754,131		$58,136	7.58	$667,360	
**5%**		**100%**	$66,834		16.44	$562,378		$87,734	12.88	$1,454,320		$47,358	8.57	$1,530,912		$60,723	7.58	$1,349,707	
		**200%**	$72,220		16.44	$1,139,780		$93,034	12.88	$2,933,511		$52,632	8.57	$3,091,912		$65,951	7.58	$2,728,599	
		**400%**	$83,034		16.44	$2,299,170		$103,648	12.88	$5,895,966		$63,148	8.57	$6,204,194		$76,378	7.58	$5,478,685	
		**20%**	$62,035		16.46	$15,392		$83,031	12.89	$38,457		$42,856	8.58	$56,394		$56,275	7.58	$82,829	
		**50%**	$63,648		16.46	$71,007		$84,618	12.89	$158,622		$44,437	8.58	$189,494		$57,852	7.58	$278,098	
**20%**		**100%**	$66,352		16.46	$164,242		$87,283	12.89	$360,432		$47,069	8.58	$411,024		$60,453	7.58	$600,244	
		**200%**	$71,754		16.46	$350,514		$92,614	12.89	$763,998		$52,333	8.58	$854,102		$65,675	7.58	$1,246,999	
		**400%**	$82,548		16.46	$722,735		$103,245	12.89	$1,568,953		$62,861	8.58	$1,740,275		$76,103	7.58	$2,538,459	
		**20%**	$60,290		16.53	Cost saving		$81,486	12.93	Cost saving		$41,846	8.61	Cost saving		$55,308	7.60	Cost saving	
		**50%**	$61,911		16.53	$3,114		$83,088	12.93	$12,082		$43,420	8.61	$28,197		$56,850	7.60	$47,443	
**70%**		**100%**	$64,615		16.53	$29,254		$85,757	12.93	$69,145		$46,058	8.61	$88,485		$59,463	7.60	$147,106	
		**200%**	$70,019		16.53	$81,493		$91,079	12.93	$182,905		$51,328	8.61	$208,939		$64,694	7.60	$346,593	
		**400%**	$80,814		16.53	$185,840		$101,741	12.93	$410,828		$61,858	8.61	$449,577		$75,108	7.60	$743,764	

* No obesity or comorbidity at baseline.

† Baseline obesity, cardiovascular disease, and non-OA musculoskeletal disease.

‡ ICER = incremental cost-effectiveness ratio in 2010 US$ per quality-adjusted life-year (QALY) gained, compared with standard TKA. “Cost saving” indicates greater QALYs achieved at a lower cost compared to standard TKA.

Remaining lifetime costs ranged from $42,186 in healthy 70–79-year-olds to $82,524 among healthy 50–59-year-olds. Innovative implants costing 20% to 400% above standard implants resulted in $446 to $20,959 increases in lifetime costs. Greater reductions in long-term failure always resulted in lower costs. Innovative implants offering a 70% reduction in long-term failure at only 20% higher cost resulted in greater QALE but lower lifetime costs than the standard implant in all four cohorts, suggesting such an implant would be cost-saving compared to standard implants if that level of TKA failure reduction could be achieved.

**Figure 2:**
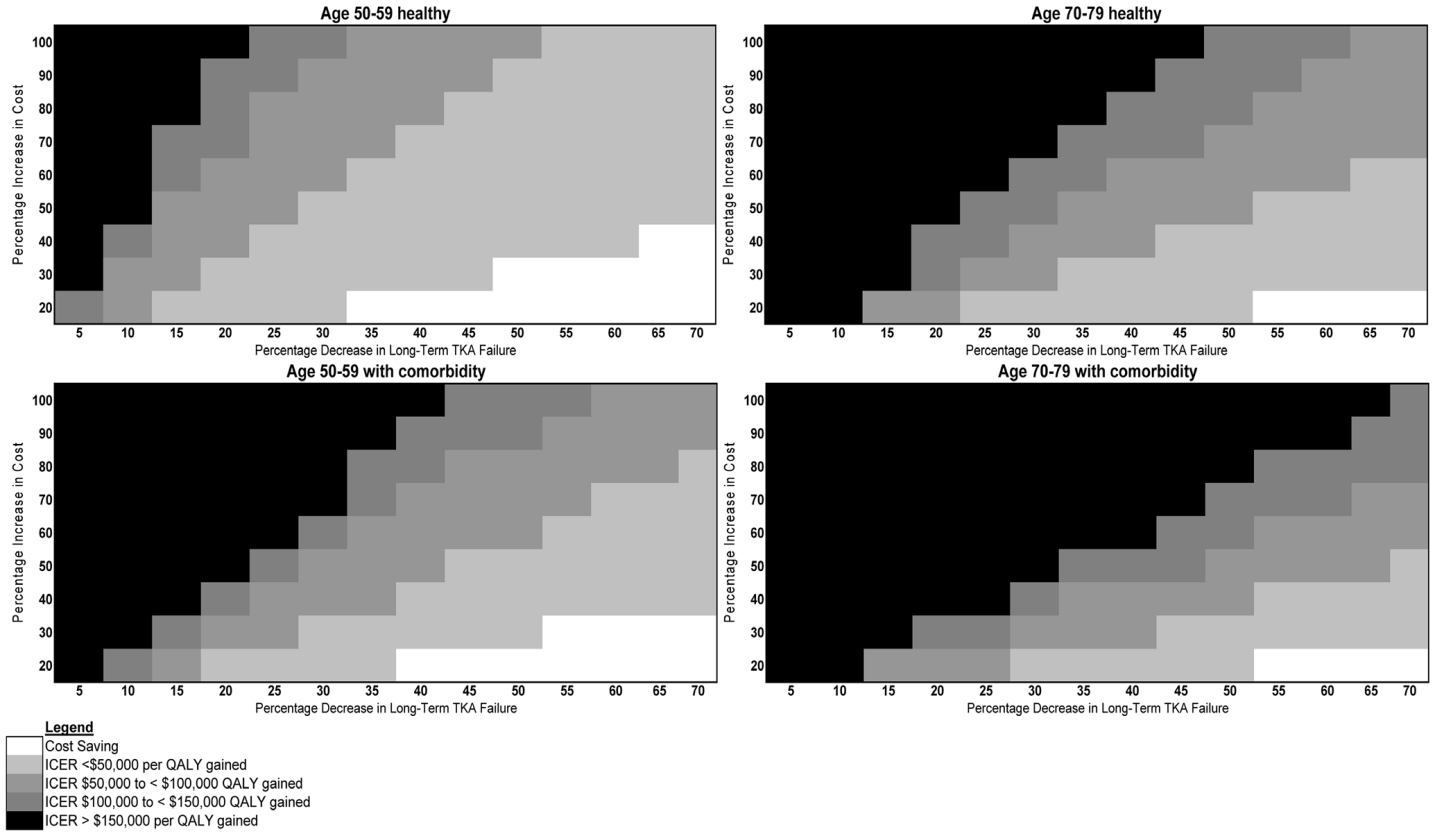
Implant cost and long-term TKA failure rate thresholds. Each shaded area represents implant cost increases (vertical axis) and failure rate reductions (horizontal axis) required to achieve a given ICER range (see Legend contained in Figure) among the four primary cohorts. ICER = incremental cost-effectiveness ratio in 2010 US$ per quality-adjusted life-year (QALY) gained, compared with standard TKA.

ICERs for innovative compared to standard implants varied with changes in both long-term implant failure and implant cost. Innovative implants costing at least three times more than a standard implant yielded ICERs above $150,000 per QALY gained in all populations except for healthy 50–59-year-olds, for which the ICER was $81,493. Implants offering a 20% reduction in long-term failure at 50% increased implant cost yielded ICERs of $71,007 per QALY gained (compared to standard TKA) among healthy 50–59-year-olds, $158,622 among 50–59-year-olds with baseline comorbidity, $189,494 among healthy 70–79-year-olds, and $278,098 among 70–79-year-olds with baseline comorbidity. More favorable ICERs were obtained when we considered a healthier, younger target population and when we assumed cheaper, more effective innovative implants (Figure 2). This trend persisted in the 60–69-year-old and 80–89-year-old cohorts (Figure 1 in the Technical Appendix).

### Sensitivity Analyses

Increasing the standard implant failure rate among healthy 50–59-year-olds produced lower ICERs for innovative implants. Tripling the standard failure rate among healthy 50–59-year-olds from 1.36% to 4.08% reduced the ICER for an implant that decreased failure by 20% at 50% greater cost from $71,007 to $22,475ska per QALY gained.

**Figure 3:**
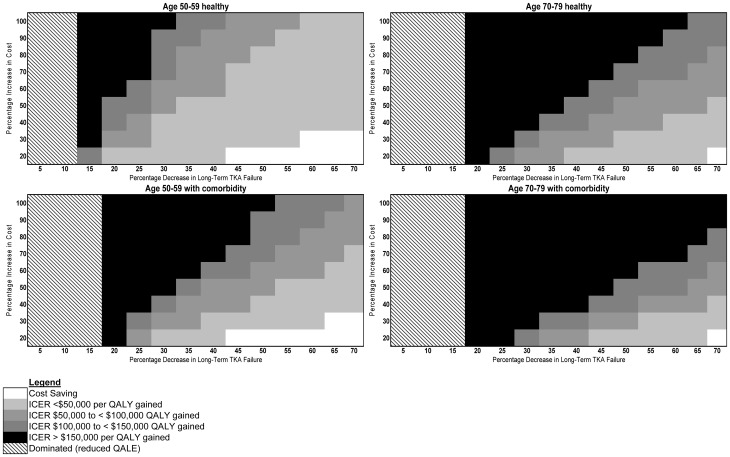
Implant cost and long-term TKA failure thresholds in a scenario where the base short-term failure rate doubles. Each shaded area represents implant cost increases (vertical axis) and failure rate reductions (horizontal axis) required to achieve a given ICER range (see Legend contained in Figure) among the four primary cohorts in 2010 US$ per quality-adjusted life year (QALY) gained, compared with standard TKA. Areas with black and white diagonal lines indicate assumptions under which innovative implants offer lower QALE for greater cost compared to standard implants (i.e., such an innovative implant is “dominated” by the standard implant).

Increasing short-term implant failure while simultaneously reducing long-term failure decreased cost-effectiveness across all cohorts (i.e., fewer innovative implants produced ICERs <$100,000 compared to standard TKA; Figure 3). Doubling short-term failure from 1.1% (the value for a standard implant) to 2.2% for an innovative implant offering a 20% reduction in long-term failure at 50% increased cost raised the ICER from $71,007 to $141,022 per QALY gained among healthy 50–59-year-olds and from $278,098 to $807,358 per QALY gained among 70–79-year-olds with comorbidity. When the short-term failure rate was doubled and reductions in long-term failure rates from innovative implants were small, innovative implants offered similar or worse QALE compared to standard implants, but at greater cost (“dominated” scenarios in Figure 3).

The impact of increasing age and comorbidity on innovative implant cost-effectiveness could be offset by either greater reductions in long-term TKA failure or improved pain relief after TKA, as might be expected with an implant that offers both technical (decreased long-term failure) and clinical (increased likelihood of pain relief) benefits (Figure 2 in the Technical Appendix). A 4% increase in the percentage of individuals experiencing pain relief after TKA reduced the ICER for an innovative implant offering a 70% reduction in long-term TKA failure at twice the cost of a standard implant, from $147,106 to $76,270 per QALY gained among 70–79-year-olds with baseline comorbidity.

Varying standard TKA implant cost (compared to the base assumption of $5,414) from $2,390 to $11,952 produced ICERs among healthy 50–59-year-olds that ranged from $82 (for lowest standard implant cost) to $92,272 (for highest standard implant cost) per QALY gained for an innovative implant that costs twice a standard implant and offers a 70% decrease in long-term failure. Performing the same analysis among 70–79-year-olds with baseline comorbidity produced ICERs ranging from $36,585 to $386,828 per QALY gained when the cost of the standard implant was similarly ranged (Figure 3 in the Technical Appendix).

## Discussion

We used TKA to demonstrate how simulation modeling can measure the value of new healthcare technologies. We quantified the impact of implant cost and longevity, and recipient age and comorbidity on innovative TKA implant cost-effectiveness. The hypothetical ability of innovative TKA implants to delay the need for revision resulted in quality of life gains as high as 0.10 QALY, consistent with other medical device innovations, such as dual-chamber pacemakers, which offer incremental QALE benefits of 0.14 QALY compared to standard pacemakers [45]. It is important to note, however, that because the revision rates of TKA with standard implants are already low, much of the population may not experience any benefit from innovative implants, while a small group of people may experience large benefits by avoiding otherwise complex revision procedures. Taken as an average across the whole population, innovative TKA implants therefore offered smaller increases in QALE than other novel therapeutics, such as dabigatran for stroke prevention in atrial fibrillation, which provided 0.42 more QALYs than warfarin, a four times greater QALE benefit than even the most optimistic innovative TKA implant in our study [46]. Further, these gains are attenuated in older populations or those with greater comorbidity that may not survive long enough to benefit from decrements in long-term TKA failure.

While there is no single accepted threshold below which a novel therapy might be considered “cost-effective”, the WHO Commission on Macroeconomics and Health offers the following guidance: interventions should be labeled “cost-effective” in a given country if their ICER is less than three times that nation’s per capita gross domestic product; interventions whose ICER is less than the per capita GDP may be labeled “highly cost-effective” [47], [48]. The GDP in the US is roughly $50,000 [49], suggesting that that innovative TKA implants offering at most a 15% reduction in long-term failure are unlikely to offer cost-effective value to a broad range of patient populations. Such implants would need to provide at least a 50% relative decrease in actual TKA failure compared with standard implants to be considered broadly cost-effective according to WHO standards.

Healthy 50–59-year-olds, those with the greatest anticipated life expectancy among our four cohorts, received the most meaningful clinical and economic benefit from decreasing the TKA failure rate. In fact, if innovative implants could be proven to reduce true long term TKA failure by at least 35% among this cohort, and if such implants cost only 20% more than existing implants, they might even offer a cost saving alternative to standard implants. This finding was accentuated when we assumed that younger, healthier TKA recipients had a higher baseline risk of revision than older less healthy individuals, as recent data suggest [43]. However, the true efficacy of innovative implants is poorly understood and, based upon recent experience with metal-on-metal implants [22], might actually lead to increased, rather than decreased TKA failure rates.

While implant cost and efficacy affected innovative TKA implant cost-effectiveness, recipient comorbidity and remaining life span also had a substantial impact. Our results suggest these factors may be even more important if innovative implants lead to increased short-term TKA failure, as one might expect with a novel technology that requires the development of specific skills and/or surgical experience to produce optimal results. When the short-term failure risk is doubled for healthy 50–59 year olds, an innovative implant with only a 10% reduction in late-term failure led to worse QALE than standard implants. The results were even less favorable in older and sicker cohorts (Figure 3). Despite yielding acceptable ICERs under some scenarios, increases in innovative implant survival offered at most a 11.32% absolute reduction in the cumulative risk of revision among healthy 50–59-year-olds and 5.83% absolute reduction among individuals aged 70–79 with baseline comorbidity.

There are limitations to our analysis. We estimated proportional decreases in TKA implant failure based upon laboratory studies of tibial and acetabular bearing surfaces [14], [19], which are unlikely to correlate precisely with clinical TKA outcomes and likely overestimate the true clinical benefit of innovative implants. However, in addition to exploring a wide range of possible effects of innovative implants on reducing TKA failure, we chose conservative estimates and assumed laboratory wear underestimates true failure our conservative estimates demonstrated that innovative biomaterials require a minimum of 50–55% reduction in true long-term failure to be considered broadly cost-effective. Published studies suggest innovative hip implants may reduce short-term *in vivo* wear rates between 40–72%, [50]–[53] but these radiographic findings may not accurately reflect clinical outcomes such as implant failure or the need for revision. Robust, long-term clinical data on TKA implants are unavailable, which is one of the advantages of simulation modeling in this area. As true TKA failure rates are unlikely to be reduced as much as short-term *ex vivo* knee or *in vivo* hip studies indicate, it is improbable that existing innovative implants offer marked economic value across all populations.

Increased activity levels among younger, healthier TKA recipients, who may therefore be at greater risk of TKA failure, may further enhance innovative implant cost-effectiveness. However, we did not model dynamic decreases in TKA failure as an individual ages, so our model likely overestimates the cost-effectiveness of innovative implants under such assumptions. In contrast, our base implant cost estimate may exceed the average cost experienced by some hospitals [54], thereby underestimating cost-effectiveness. Since TKA cost-effectiveness is sensitive to implant cost, TKA innovations may be more or less cost-effective depending upon the implant cost each hospital pays, producing variability in value according to the negotiated cost [21]. While we did not assess the impact of surgical technique on cost-effectiveness, we did examine the possibility that innovative technology might offer greater short-term failure due to the potential for increased technical complexity associated with novel devices. We also did not consider that the decision, from a patient’s or society’s perspective, to adopt an innovative implant today is to forgo the option of an even better implant tomorrow. However, similar limitations are likely to affect the evaluation of any innovative medical device and can be addressed by sensitivity analyses to assess the impact of data uncertainty.

The FDA 510(k) clearance process has been criticized for insufficient safety oversight and suppressing innovation through incentivizing incremental gains [44], [55]. Limited oversight of medical device approval combined with economic and other incentives serve to speed technology adoption [56]. However, recent data demonstrate expedited approval is associated with increased rates of high-risk product recalls [57]. Our analyses suggest that small, incremental improvements in device longevity may have little to no effect on QALE and cost-effectiveness, further supporting the Institute of Medicine’s recent call for redesigning the 510(k) approval process [58]. Furthermore, extensive recent data demonstrate that not all innovative implant technologies will lead to actual improvements in longevity, and some, such as metal-on-metal hip implants, may even lead to worse long-term outcomes [22], [59]–[62]. Innovative implants providing no clinical benefit at an added cost would not be cost-effective. Decisions as to the marketing, use and reimbursement of medical devices are currently being made without robust outcomes data to guide decision making. Our approach serves as a model for evaluating a wide range of innovative technologies.

Because innovative implant technologies are released at about a rate of 30 per year [11], surgeons and hospitals are often faced with choices of whether to use specific innovative devices. These devices may utilize one or more innovative technologies, such as cementless fixation, rotating or sliding bearing inserts, novel biomaterials, or gender-specific designs. For several of these technologies, it is not clear whether they offer a significant improvement in clinical outcomes over existing prostheses [63]–[65]. However, as data from joint registries and head-to-head trials uncover which specific implants can offer improved clinical outcomes and/or reduced rates of revision, our model allows decision makers to combine these outcomes data with the implant’s marketed cost to determine whether such a device would be cost-effective in particular patient populations.

While it is difficult to predict the benefit of an innovative TKA implant to any one individual, the incremental benefit to society appears modest, and the excess cost of using innovative implants indiscriminately across populations could be substantial. Compared to per capita healthcare expenditures among industrialized nations, TKA alone is responsible for $3 billion in increased US spending [66]. Targeting implants to specific populations based on their expected clinical benefit may offer significant savings. Moreover, TKA is representative of many medical devices, ranging from hip and spinal implants, plates, screws and other orthopedic hardware, to cataract and ophthalmologic implants, which offer improvements in quality of life rather than survival benefits. This work has implications for the development and adoption of any medical device offering long-term clinical benefit at a greater initial cost. Our analysis demonstrates that small decreases in long-term device failure can provide clinical and economic value under certain circumstances, but these innovations may not offer equal benefit in all populations. In addition, many innovative technologies offer improved long-term outcomes at the expense of increased short-term complications due to increased complexity of the procedure and/or the technical skill required to optimally implement such advances. Our data suggest that even if innovative implants lead to some increases in short-term complications, the impact on overall cost-effectiveness will be small in situations where innovative devices offer substantial benefit over standard devices in reducing long term failure. In situations where innovative implants are associated with lowering rather than increasing short-term complications, cost-effectiveness will likely be more favorable.

These results also provide important insights regarding the costs and benefits associated with the diffusion of technology into clinical practice. The modeling approach presented here can and should focus and improve device development, identify optimal populations for testing novel technologies, and provide physicians, insurers and patients detailed information on the clinical benefits expected for a given investment. The complex trade-offs between short- and long-term health and economic consequences of technological innovation cannot easily be captured by even the most sophisticated randomized trials. Model-based evaluations such as those presented here may provide important insights for evaluating medical device innovation and merit consideration as a standard component of the evaluation process.

## Supporting Information

Technical Appendix S1 **Additional information to supplement the methods and results sections of this manuscript.** It further explains input parameters utilized to model total knee Arthroplasty as a treatment for knee osteoarthritis and provides supplemental results including a number of sensitivity analyses not present in the manuscript.
(DOC)

## References

[1] Suter LG, Paltiel AD, Rome BN, Solomon DH, Golovaty I, et al (2011) Medical device innovation–is “better” good enough? New England Journal of Medicine 365: 1464–1466. 22010911 10.1056/NEJMp1109285 PMC3570242

[2] Healthcare Cost and Utilization Project (HCUP) (2008) Nationwide Inpatient Sample (NIS). Agency for Healthcare Research and Quality. Available: http://hcupnet.ahrq.gov/. Accessed 2012 Jun 9, 2012.

[3] Stranges E, Russo CA, Friedman B (2009) Procedures with the Most Rapidly Increasing Hospital Costs, 2004–2007: Statistical Brief #82. Healthcare Cost and Utilization Project (HCUP) Statistical Briefs.

[4] Hawker G, Wright J, Coyte P, Paul J, Dittus R, et al (1998) Health-related quality of life after knee replacement. Journal of Bone & Joint Surgery - American Volume 80: 163–173. 10.2106/00004623-199802000-00003 9486722

[5] Heck DA, Robinson RL, Partridge CM, Lubitz RM, Freund DA (1998) Patient outcomes after knee replacement. Clinical Orthopaedics & Related Research: 93–110. 10.1097/00003086-199811000-00015 9917673

[6] Callahan CM, Drake BG, Heck DA, Dittus RS (1994) Patient outcomes following tricompartmental total knee replacement. A meta-analysis. Journal of the American Medical Association 271: 1349–1357. 8158821

[7] Katz JN, Mahomed NN, Baron JA, Barrett JA, Fossel AH, et al (2007) Association of hospital and surgeon procedure volume with patient-centered outcomes of total knee replacement in a population-based cohort of patients age 65 years and older. Arthritis & Rheumatism 56: 568–574. 17265491 10.1002/art.22333

[8] Losina E, Walensky RP, Kessler CL, Emrani PS, Reichmann WM, et al (2009) Cost-effectiveness of total knee arthroplasty in the United States: patient risk and hospital volume. Archives of Internal Medicine 169: 1113–1121. 19546411 10.1001/archinternmed.2009.136 PMC2731300

[9] Crowninshield RD, Rosenberg AG, Sporer SM (2006) Changing Demographics of Patients with Total Joint Replacement. Clinical Orthopaedics & Related Research 443: 266–272. 16462450 10.1097/01.blo.0000188066.01833.4f

[10] Kurtz SM, Lau E, Ong K, Zhao K, Kelly M, et al (2009) Future young patient demand for primary and revision joint replacement: national projections from 2010 to 2030. Clinical Orthopaedics & Related Research 467: 2606–2612. 19360453 10.1007/s11999-009-0834-6 PMC2745453

[11] Losina E, Thornhill TS, Rome BN, Wright J, Katz JN (2012) The dramatic increase in total knee replacement utilization rates in the United States cannot be fully explained by growth in population size and the obesity epidemic. J Bone Joint Surg Am 94: 201–207. 22298051 10.2106/JBJS.J.01958 PMC3262184

[12] Search of 510(k) Premarket Notification database for knee prosthesis product codes from January 1, 2000 through December 31, 2010. U.S. Food and Drug Administration. Available: http://www.accessdata.fda.gov/scripts/cdrh/cfdocs/cfpmn/pmn.cfm. Accessed 2012 Jun 23.

[13] McEwen HM, Barnett PI, Bell CJ, Farrar R, Auger DD, et al (2005) The influence of design, materials and kinematics on the in vitro wear of total knee replacements. Journal of Biomechanics 38: 357–365. 15598464 10.1016/j.jbiomech.2004.02.015

[14] Stryker X3 Advanced Bearing Technology. Stryker Corporation. Available: http://www.stryker.com/en-us/products/Orthopaedics/KneeReplacement/X3AdvancedBearingTechnology/006589. Accessed 2012 Jun 9.

[15] Muratoglu OK, Bragdon CR, Jasty M, O’Connor DO, Van Knoch RS, et al (2004) Knee-simulator testing of conventional and cross-linked polyethylene tibial inserts. Journal of Arthroplasty 19: 887–897. 15483806 10.1016/j.arth.2004.03.019

[16] Muratoglu OK, Bragdon CR, O’Connor DO (2002) Perinchief RS, Jasty M, et al (2002) Aggressive wear testing of a cross-linked polyethylene in total knee arthroplasty. Clinical Orthopaedics & Related Research 404: 89–95. 10.1097/00003086-200211000-00015 12439243

[17] Muratoglu OK, Mark A, Vittetoe DA, Harris WH, Rubash HE (2003) Polyethylene damage in total knees and use of highly crosslinked polyethylene. Journal of Bone & Joint Surgery - American Volume 85: S7–S13. 10.2106/00004623-200300001-00003 12540663

[18] Muratoglu OK, Rubash HE, Bragdon CR, Burroughs BR, Huang A, et al (2008) Simulated normal gait wear testing of a highly crosslinked polyethylene tibial insert. Journal of Arthroplasty 22: 435–444. 10.1016/j.arth.2006.07.014 17400100

[19] The first knee tested for 30 simulated years of wear performance. Smith & Nephew. Available: http://global.smith-nephew.com/us/patients/LEGION_Knee_with_VERILAST_15431.htm. Accessed 2012 Jun 9.

[20] Frost & Sullivan (2005) U.S. & Asian Markets for Orthopedic Joint Replacement. Market Research Report (Product Code R1–2948).

[21] MD Buyline Inc. (2008) The Impact of Consumables: Orthopedic Joint Replacement.

[22] Smith AJ, Dieppe P, Vernon K, Porter M, Blom AW (2012) Failure rates of stemmed metal-on-metal hip replacements: analysis of data from the National Joint Registry of England and Wales. Lancet 379: 1199–1204. 22417410 10.1016/S0140-6736(12)60353-5

[23] Losina E, Walensky RP, Holt HL, Gerlovin H, Reichmann WM, et al (2011) Impact of Obesity and Knee Osteoarthritis on Morbidity and Mortality in Older Americans. Annals of Internal Medicine 154: 217–226. 21320937 10.1059/0003-4819-154-4-201102150-00001 PMC3260464

[24] Holt HL, Katz JN, Reichmann WM, Gerlovin H, Wright EA, et al (2010) Forecasting the burden of advanced knee osteoarthritis over a 10-year period in a cohort of 60–64 year-old US adults. Osteoarthritis & Cartilage 19: 44–50. 20955807 10.1016/j.joca.2010.10.009 PMC3010490

[25] Kurtz SM (2004) The UHMWPE Handbook. Ultra-High Molecular Weight Polyethylene in Total Joint Replacement. San Diego: Elsevier Academic Press. 396 p.

[26] Skinner J, Zhou W, Weinstein J (2006) The influence of income and race on total knee arthroplasty in the United States. Journal of Bone & Joint Surgery - American Volume 88: 2159–2166. 10.2106/JBJS.E.00271 PMC2749956 17015592

[27] Katz JN, Barrett J, Mahomed NN, Baron JA, Wright RJ, et al (2004) Association Between Hospital and Surgeon Procedure Volume and the Outcomes of Total Knee Replacement. Journal of Bone & Joint Surgery - American Volume 86: 1909–1916. 10.2106/00004623-200409000-00008 15342752

[28] Reichmann WM, Katz JN, Kessler CL, Jordan JM, Losina E (2009) Determinants of self-reported health status in a population-based sample of persons with radiographic knee osteoarthritis. Arthritis Rheum 61: 1046–1053. 19644892 10.1002/art.24839 PMC2747662

[29] Taylor DC, Pandya A, Thompson D, Chu P, Graff J, et al (2009) Cost-effectiveness of intensive atorvastatin therapy in secondary cardiovascular prevention in the United Kingdom, Spain, and Germany, based on the Treating to New Targets study. European Journal of Health Economics 10: 255–265. 18800232 10.1007/s10198-008-0126-1

[30] Melnikow J, Birch S, Slee C, McCarthy TJ, Helms LJ, et al (2008) Tamoxifen for breast cancer risk reduction: impact of alternative approaches to quality-of-life adjustment on cost-effectiveness analysis. Medical Care 46: 946–953. 18725849 10.1097/MLR.0b013e318179250f

[31] Weaver M, Krieger J, Castorina J, Walls M, Ciske S (2001) Cost-effectiveness of combined outreach for the pneumococcal and influenza vaccines. Archives of Internal Medicine 161: 111–120. 11146707 10.1001/archinte.161.1.111

[32] Fisman DN, Reilly DT, Karchmer AW, Goldie SJ (2001) Clinical effectiveness and cost-effectiveness of 2 management strategies for infected total hip arthroplasty in the elderly. Clinical Infectious Diseases 32: 419–430. 11170950 10.1086/318502

[33] Centers for Disease Control and Prevention (CDC) (2006) 2005–2006 National Health and Nutrition Examination Survey (NHANES) Data. Hyattsville, MD: National Center for Health Statistics (NCHS), U.S. Department of Health and Human Services.

[34] Centers for Disease Control and Prevention (CDC) (2008) 2007–2008 National Health and Nutrition Examination Survey (NHANES) Data. Hyattsville, MD: National Center for Health Statistics (NCHS), U.S. Department of Health and Human Services.

[35] Lee DW, Meyer JW, Clouse J (2001) Implications of Controlling for Co-morbid Conditions in Cost-of-Illness Estimates: A Case Study of Osteoarthritis from a Managed Care Perspective. Value in Health 4: 329–334. 11705300 10.1046/j.1524-4733.2001.44012.x

[36] (2008) Red Book: Pharmacy’s Fundamental Reference: 2008 Edition. Montvale, NJ: Thomson Healthcare.

[37] Mendenhall S (2004) Hip and knee implant prices increase 9%. OR Manager 20: 32. 15521340

[38] Robinson JC, Pozen A, Tseng S, Bozic KJ (2012) Variability in Costs Associated with Total Hip and Knee Replacement Implants. J Bone Joint Surg Am. 10.2106/JBJS.K.00355 22878562

[39] (2008) Physician Fee Schedule Search. Center for Medicare and Medicaid Services (CMS). Available: http://www.cms.gov/apps/physician-fee-schedule/overview.aspx. Accessed 2012 Jun 9.

[40] Teeny SM, York SC, Mesko JW, Rea RE (2003) Long-Term Follow-Up Care Recommendations after Total Hip and Knee Arthroplasty: results of the American Association of Hip and Knee Surgeons’ member survey. Journal of Arthroplasty 18: 954–962. 14658097 10.1016/j.arth.2003.09.001

[41] Gold MR, Siegel JE, Russell LB, Weintstein MC (1996) Cost Effectiveness in Health and Medicine. New York: Oxford University Press.

[42] Bozic KJ, Morshed S, Silverstein MD, Rubash HE, Kahn JG (2006) Use of cost-effectiveness analysis to evaluate new technologies in orthopaedics. The case of alternative bearing surfaces in total hip arthroplasty. Journal of Bone & Joint Surgery - American Volume 88: 706–714. 10.2106/JBJS.E.00614 16595459

[43] Paxton EW, Namba RS, Maletis GB, Khatod M, Yue EJ, et al (2010) A prospective study of 80,000 total joint and 5000 anterior cruciate ligament reconstruction procedures in a community-based registry in the United States. Journal of Bone & Joint Surgery - American Volume 92 Suppl 2117–132. 10.2106/JBJS.J.00807 21123596

[44] Curfman GD, Redberg RF (2011) Medical devices–balancing regulation and innovation. New England Journal of Medicine 365: 975–977. 21830959 10.1056/NEJMp1109094

[45] Rinfret S, Cohen DJ, Lamas GA, Fleischmann KE, Weinstein MC, et al (2005) Cost-effectiveness of dual-chamber pacing compared with ventricular pacing for sinus node dysfunction. Circulation 111: 165–172. 15630030 10.1161/01.CIR.0000151810.69732.41

[46] Freeman JV, Zhu RP, Owens DK, Garber AM, Hutton DW, et al (2011) Cost-effectiveness of dabigatran compared with warfarin for stroke prevention in atrial fibrillation. Ann Intern Med 154: 1–11. 21041570 10.7326/0003-4819-154-1-201101040-00289

[47] World Health Organization (2001) Macroeconomics and Health: Investing in Health for Economic Development.

[48] World Health Organization CHOosing Interventions that are Cost Effective (WHO-CHOICE). Available: http://www.who.int/choice/costs/CER_thresholds/en/index.html. Accessed 2013 Feb 15.

[49] International Monetary Fund (2009) World economic outlook database. World economic and financial surveys. Available: http://www.imf.org/external/pubs/ft/weo/2012/02/weodata/index.aspx. Accessed 2013 Feb 14.

[50] Glyn-Jones S, McLardy-Smith P, Gill HS, Murray DW (2008) The creep and wear of highly cross-linked polyethylene: a three-year randomised, controlled trial using radiostereometric analysis. Journal of Bone & Joint Surgery - British Volume 90: 556–561. 10.1302/0301-620X.90B5.20545 18450618

[51] Glyn-Jones S, Isaac S, Hauptfleisch J, McLardy-Smith P, Murray DW, et al (2008) Does highly cross-linked polyethylene wear less than conventional polyethylene in total hip arthroplasty? A double-blind, randomized, and controlled trial using roentgen stereophotogrammetric analysis. Journal of Arthroplasty 23: 337–343. 18358369 10.1016/j.arth.2006.12.117

[52] Heisel C, Silva M, Dela Rosa MA, Schmalzried TP (2004) Short-term in vivo wear of cross-linked polyethylene. Journal of Bone & Joint Surgery - American Volume 86: 748–751. 10.2106/00004623-200404000-00012 15069139

[53] Martell JM, Verner JJ, Incavo SJ (2003) Clinical performance of a highly cross-linked polyethylene at two years in total hip arthroplasty: a randomized prospective trial. Journal of Arthroplasty 18: 55–59. 10.1016/s0883-5403(03)00341-3 14560412

[54] Anonymous (2009) Market grows, but prices for total knee components decline in 2009 (PRICE SURVEY). Hospital Materials Management 34: 9–11. 19374140

[55] Challoner DR, Vodra WW (2011) Medical devices and health–creating a new regulatory framework for moderate-risk devices. New England Journal of Medicine 365: 977–979. 21830961 10.1056/NEJMp1109150

[56] Mundy A (2009) FDA to Review Medical-Device Approval. The Wall Street Journal. New York, NY: The Wall Street Journal.

[57] Zuckerman DM, Brown P, Nissen SE (2011) Medical Device Recalls and the FDA Approval Process. Archives of Internal Medicine 171: 1006–1011. 21321283 10.1001/archinternmed.2011.30

[58] Institute of Medicine (IOM) (2011) Medical Devices and the Public’s Health: The FDA 510(k) Clearance Process at 35 Years. Washington, DC: National Acadamies Press.

[59] Anand R, Graves SE, de Steiger RN, Davidson DC, Ryan P, et al (2011) What is the benefit of introducing new hip and knee prostheses? J Bone Joint Surg Am 93 Suppl 351–54. 22262424 10.2106/JBJS.K.00867

[60] Healy WL, Iorio R (2007) Implant selection and cost for total joint arthroplasty: conflict between surgeons and hospitals. Clin Orthop Relat Res 457: 57–63. 17242613 10.1097/BLO.0b013e31803372e0

[61] Kalisvaart MM, Pagnano MW, Trousdale RT, Stuart MJ, Hanssen AD (2012) Randomized clinical trial of rotating-platform and fixed-bearing total knee arthroplasty: no clinically detectable differences at five years. J Bone Joint Surg Am 94: 481–489. 22437996 10.2106/JBJS.K.00315

[62] Luo R, Brekke A, Noble PC (2012) The Financial Impact of Joint Registries in Identifying Poorly Performing Implants. J Arthroplasty. 10.1016/j.arth.2012.03.046 22682045

[63] Gandhi R, Tsvetkov D, Davey JR, Mahomed NN (2009) Survival and clinical function of cemented and uncemented prostheses in total knee replacement: a meta-analysis. J Bone Joint Surg Br 91: 889–895. 19567852 10.1302/0301-620X.91B7.21702

[64] Park JW, Kim YH (2011) Simultaneous cemented and cementless total knee replacement in the same patients: a prospective comparison of long-term outcomes using an identical design of NexGen prosthesis. J Bone Joint Surg Br 93: 1479–1486. 22058298 10.1302/0301-620X.93B11.27507

[65] Wylde V, Learmonth I, Potter A, Bettinson K, Lingard E (2008) Patient-reported outcomes after fixed- versus mobile-bearing total knee replacement: a multi-centre randomised controlled trial using the Kinemax total knee replacement. J Bone Joint Surg Br 90: 1172–1179. 18757956 10.1302/0301-620X.90B9.21031

[66] McKinsey Global Institute (2008) Accounting for the cost of US health care: A new look at why Americans spend more. McKinsey & Company. 59 p.

