# Supplementary material for: Placing a Price on Medical Device Innovation: The Example of Total Knee Arthroplasty
Source: PLoS One. 2013 May 6;8(5):e62709. doi: 10.1371/journal.pone.0062709 (PMC3646021; doi:10.1371/journal.pone.0062709)
Supplement: Technical Appendix S1 — Additional information to supplement the methods and results sections of this manuscript. It further explains input parameters utilized to model total knee Arthroplasty as a treatment for knee osteoarthritis and provides supplemental results including a number of sensitivity analyses not present in the manuscript. (DOC) [file pone.0062709.s001.doc]

Placing a Price on Medical Device Innovation:

The Example of Total Knee Arthroplasty

TECHNICAL APPENDIX

This Technical Appendix supplements the methods and results sections of the manuscript entitled “Placing a Price on Medical Device Innovation: The Example of Total Knee Arthroplasty.” The goals of this Technical Appendix are: 1) to further explain the input parameters utilized to model total knee arthroplasty (TKA) as a treatment for knee osteoarthritis (OA) (Part I); and 2) to provide supplemental results and explain a number of sensitivity analyses not presented in the manuscript (Part II).

# Part I. Input Parameters for Modeling Total Knee Arthroplasty as a Treatment for Knee OA

The OAPol Model is a validated state-transition model that utilizes Monte Carlo simulations to determine annual health states for a hypothetical cohort of individuals over the course of their lifetime. Details on the overall structure of the OAPol Model and the determination of yearly health states have been previously published.

In addition to estimating yearly health states, the OAPol model allows individuals to undergo treatment for knee OA symptoms. Treatments affect cost, quality, and quantity of life, and each is associated with its own set of probabilistic parameters to define utilization, efficacy, and adverse events. For this analysis, all individuals underwent primary total knee arthroplasty (TKA), and those individuals who experienced a failure could go on to receive revision TKA. The sections below describe the model input parameters used to model primary and revision TKA.

## I.A. Eligibility, Offer, and Acceptance

**All individuals in this analysis were initiated with 100% eligibility, offer, and acceptance of primary TKA. However, for revision TKA we** endeavored to model “real” clinical practice, where not all primary TKA failures present immediately for medical attention and not all people offered a revision surgery accept. **Only a mechanical prosthesis failure (i.e., technical failure) of primary TKA qualified an individual for revision TKA, but the presence of pain resulted in a greater likelihood of being offered revision surgery by a physician (50% with pain versus 10% without pain).** Additionally, of those individuals offered a revision surgery, only 70% accepted revision. Individuals who refused TKA revision when initially offered were offered again each subsequent year, but the acceptance rate was much lower (21%). These probabilities were derived through a calibration exercise to reproduce the estimated 2.95% cumulative rate of revision TKA among Medicare beneficiaries, according to published literature.

## I.B. Efficacy

**We assumed that TKAs would fail at a constant annual rate, based upon epidemiologic data from the Swedish Knee Arthroplasty Register supporting a linear technical failure rate after the first year as well as laboratory data suggesting long-term polyethylene wear is also linear after the first year . Symptomatic efficacy rates were derived based on survey data from a cohort of Medicare patients undergoing primary or revision TKA . First-year pain failure rates were based on the percent of patients who responded that their knee pain was never resolved by primary (13.8%) and revision (25.7%) TKA. Annual rates of developing pain (after an initially successful TKA) were derived based on the percent of individuals experiencing knee pain one and two years after receiving a primary TKA using the following equation:**

Let LtF = The annual percent of individuals whose TKA ceases to provide pain relief

T_1_ = time point 1 in years since TKA

T_2_ = time point 2 in years since TKA

*PnT_1_* = the percent of persons experiencing pain relief at time point 1

*PnT_2_* = the percent of persons experiencing pain relief at time point 2

$LtF=\left(\frac{PnT_{1}}{PnT_{2}}\right)^{\frac{1}{T_{2}-T_{1}}}$ [1]

## I.C. Adverse Events

Both medical and surgical adverse events (AEs) and their related mortalities were considered for primary and revision TKA. **Post-operative medical AEs in the model included myocardial infarction, pneumonia leading to hospitalization, and pulmonary embolus; surgical AEs included prosthetic joint infection leading to prosthesis removal and rehabilitation.** Medical toxicities resulted in an associated cost and decreased quality of life utility for the year following surgery, but they had no long-term affect on an individual’s course of treatment. Surgical toxicities similarly affected cost and quality of life, but they also resulted in a TKA failure that qualified for surgical revision. The likelihoods for all complications were derived from a sample of 2000 Medicare claims . Surgical mortality rates were calculated separately from AEs.

Quality of life utility estimates for TKA-related AEs were derived from published literature . All patients experienced six months of mild disutility to reflect the post-operative recovery period. Individuals could also experience any of the possible medical AEs, resulting in one month of severe disutility plus five months of mild recovery disutility, or a prosthetic joint infection which resulted in six months of severe disutility. Toxicity costs were derived from the Healthcare Cost and Utilization Project’s (HCUP) Nationwide Inpatient Sample .

## I.D. Costs

Details on the derivation of total costs for primary and revision TKA have been previously published . These costs were composed of three components: hospital costs, physician fees, and rehabilitation costs. All values were derived from Medicare data and stratified by patient risk categories and hospital volume . The resulting inclusive costs of primary and revision TKA, converted to 2010 US dollars, were $23,903 and $28,195, respectively.

Follow-up costs for TKA were assumed to stem from regular physician visits and imaging. The costs of a physician visit, an x-ray, and an MRI were obtained from 2008 Medicare data . All follow-up visits were assumed to include an x-ray and 10% of visits included an MRI of the contralateral knee, yielding an average visit cost of $155. The frequency of follow-up visits was estimated to be 0.668 (about 2 visits every 3 years) based on a calculated average from a national survey of orthopedists’ recommendations . The follow-up cost for primary and revision TKA was estimated at $103 per year.

## I.E. Data Regarding Innovative TKA Implants

We used the example of ultra-high molecular weight polyethylene to represent a hypothetical standard implant and compared this to highly crossed-linked polyethylene or other innovative tibial components, such as innovative biomaterials or rotating prostheses (i.e., innovative implant).

Short-term, *in vivo* studies of highly cross-linked polyethylene hip implants have noted between 40% and 72% reduction in radiographic wear compared with standard polyethylene but these reductions are captured by sensitive measurement or imaging techniques and may not accurately reflect clinical outcomes such as implant failure or the need for revision. Similar wear reduction has been shown in knees, but mainly through *ex vivo* simulations . Unpublished *ex vivo* data from prosthetic manufacturers report implant wear reduction as high as 81% or 97% for second generation highly cross-linked polyethylene or other dual component systems. In the analyses presented in the main manuscript, we conservatively assumed up to 70% reductions in long-term technical failure rates from innovative implants, as reductions in laboratory wear rates are unlikely to translate directly into equivalent clinical failure rates.

# Part II. Additional Sensitivity Analyses

## II.A. Demographics and Comorbidity

We studied two additional age cohorts in sensitivity analyses (60-69 and 80-89 years). Results for the effect of innovative implants on QALE, costs, and cost-effectiveness for 60-69 year and 80-89 cohorts are provided in Appendix Figure 1. As with 50-59- and 70-79-year-olds, decreasing long-term TKA failure (and therefore revision) improved cost-effectiveness of innovative implants compared to standard TKA, while increasing innovative implant costs reduced cost-effectiveness.

Given that our assumptions for sex, race, and ethnicity distributions were based on Medicare beneficiaries , we performed sensitivity analyses to ensure that these values did not significantly alter results, especially for younger cohorts who may not be accurately represented by the Medicare population. We based our sensitivity analyses on demographic data from a nationally-representative cohort of TKR recipients . Sensitivity analyses varying sex, race, and ethnicity distributions demonstrated no impact on innovative implant cost-effectiveness.

A*ppendix Figure 1: Implant cost and long-term TKA failure rate thresholds[[1]](#footnote-2)*

## II.B. TKA Revision, Offer, and Acceptance Rates

To estimate the societal benefits and costs of perfect TKA implementation (i.e., every failed TKA is recognized and offered revision, and every offer for revision is accepted), we considered an “ideal” scenario (in contrast to our base case analysis “real” scenario, see Section I.A. above), where all TKA failures were observed and offered revision TKA and all offers were accepted.

The difference in the ICER between an “ideal” scenario and our “real-life” scenario only qualitatively altered 4% of innovative implants (i.e., flipped the ICER from < $100,000 per QALY gained to > $100,000 per QALY gained or vice versa). The largest changes were found among the extreme cost-effectiveness ratios – those ICERs <$25,000 per QALY gained and those >$250,000 per QALY gained.

## II.C. Quality of Life

We have shown previously that standard **TKA cost-effectiveness is robust to variability in the risk of surgical complications and efficacy assumptions across a variety of patient cohorts, but highly sensitive to the quality of life gains after TKA and somewhat sensitive to TKA cost .** Similarly, innovative implant cost-effectiveness was sensitive to variations in TKA outcomes (the proportion of population achieving pain relief after TKA). The effect of simultaneously varying implant cost, long-term failure and the proportion of individuals achieving pain relief after TKA with innovative versus standard implants on innovative implant cost-effectiveness is summarized in Appendix Figure 2. In order to illustrate how alterations in the proportion of individuals achieving pain relief after TKA with innovative implants affected innovative implant cost-effectiveness, we selected two of the four patient cohorts (healthy 50-59 year olds, for whom innovative implants are almost universally cost-effective, and 70-79 year olds with baseline comorbidity, for whom innovative implants are only selectively cost-effective, according to commonly accepted thresholds). The zero point on the horizontal axis represents the proportion of patients experiencing pain relief after standard TKA. Each deviation from zero on the horizontal axis represents an increase or decrease in this proportion. Among the healthy, younger cohort, a 4% decrease in the proportion of patients experiencing pain relief after TKA with an innovative implant offering a 70% reduction in long-term failure and a 100% implant cost increase doubles the ICER (from $29,254 to $54,778 per QALY gained, compared to standard implants). Among the older cohort with baseline comorbidity, a 4% increase in the proportion of patients experiencing pain relief after TKA with a similar innovative implant yields an ICER of $76,270 per QALY gained (compared to $147,106 per QALY gained without this increase).

*Appendix Figure 2: The impact of the proportion of individuals achieving pain relief after TKA with innovative versus standard implants on innovative implant cost-effectiveness[[2]](#footnote-3)*

## II.D. Implant Cost

Though data is readily available to estimate the total cost of primary and revision TKA , the cost specific to knee implants is not well documented. For our analysis, we assumed a standard implant cost of $5,414, representing approximately 23% of the total cost of primary TKA , Recent data suggest that this number may vary widely according to geographic region, hospital size, and TKA volume, as well as implant type , Given that we present cost increases for innovations as a percentage of this standard cost, a change in this baseline value affects the cost of all innovative implants presented. For example, an innovative implant that increases cost by 100% would represent a less significant absolute cost increase if the standard implant cost were lower than $5,414 and a more significant increase if the cost were higher than $5,414. Consequently, we conducted a sensitivity analysis to show the effects of varying the standard implant cost. We held the total cost of TKA constant at $23,903 and varied standard implant cost from 10% to 50% of this total ($2,390 to $11,952). Reducing the proportional cost of the standard implant from 23% to only 10% improved cost-effectiveness (ICERs <$50,000 per QALY gained) among all age and comorbidity cohorts for an innovative implant offering a 70% reduction in late failure at a 100% increase in cost compared with the standard implant (see Appendix Figure 3). Similarly, increasing the proportional cost of the standard implant to 50% of total TKA cost produced ICERs >$100,000 per QALY among all cohorts except healthy 50-59-year-olds.

*Appendix Figure 3: Impact of varying standard implant[[3]](#footnote-4) cost on overall cost-effectiveness for an innovative implant representing a 70% decrease in long-term failure and a 100% increase in cost*

**References**

1. Each shaded area represents the implant cost increases (vertical axis) and failure rate reductions (horizontal axis) required to achieve a given incremental cost-effectiveness ratio (ICER) range (see Legend above) among the four cohorts. [↑](#footnote-ref-2)
2. All data points shown are for an innovative implant offering a 70% reduction in long-term TKA failure at twice the cost of a standard implant (100% cost increase). Percentages noted on the horizontal axis denote relative deviations from the proportion of individuals experiencing pain relief after standard TKA (86.2%). ICER = Incremental cost-effectiveness ratio, in 2010 US$ per QALY gained, compared with TKA using a standard implant. QALY = quality-adjusted life-year gained. [↑](#footnote-ref-3)
3. The vertical, dashed line represents the standard implant cost of $5,414 used for our base case analysis. [↑](#footnote-ref-4)
